# Supplementary figures and images for: Automated, high-throughput quantification of EGFP-expressing neutrophils in zebrafish by machine learning and a highly-parallelized microscope
Source: PLoS One. 2023 Dec 7;18(12):e0295711. doi: 10.1371/journal.pone.0295711 (PMC10703246; doi:10.1371/journal.pone.0295711)

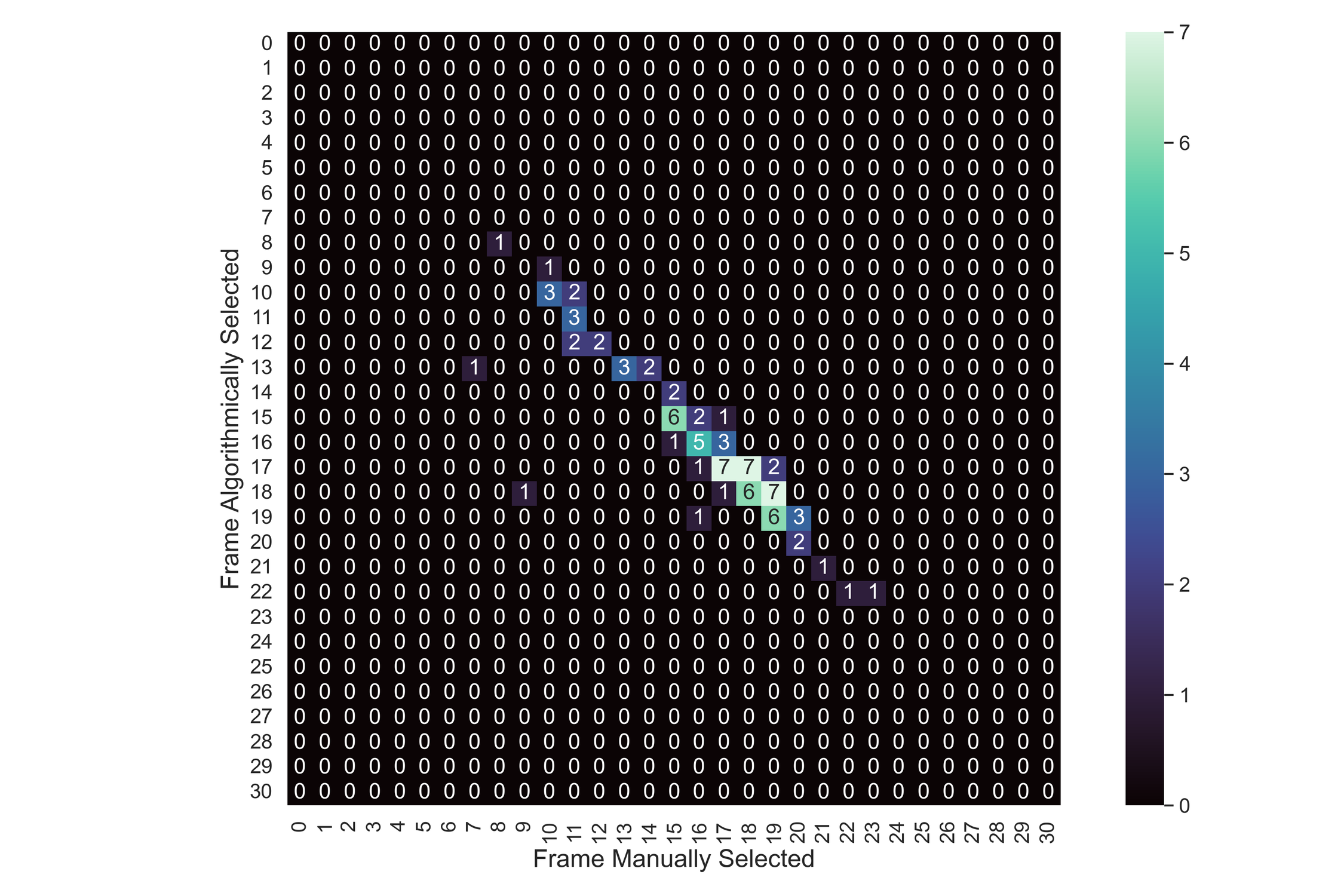

Supplement: S1 Fig — Confusion matrix showing the correlation between manual and algorithmic selection of best-focus frames. The fish in each frame is segmented by a machine learning segmentation model and the variance of the Laplacian of this region is computed and maximized to select the best focus frame from each Z-stack. When manual selection matches algorithmic selection, counts lie along the diagonal from top left to bottom right. 54% of selections match exactly between manual and algorithmic selection while 92% of the algorithmic frame selections are within one frame of the manually selected. Data represented here is from one 96-well plate and suggests that many extra z-slices were acquired than were needed because only the center ~1.5 mm were the in-focus frames of interest. (TIF) [file pone.0295711.s001.tif]

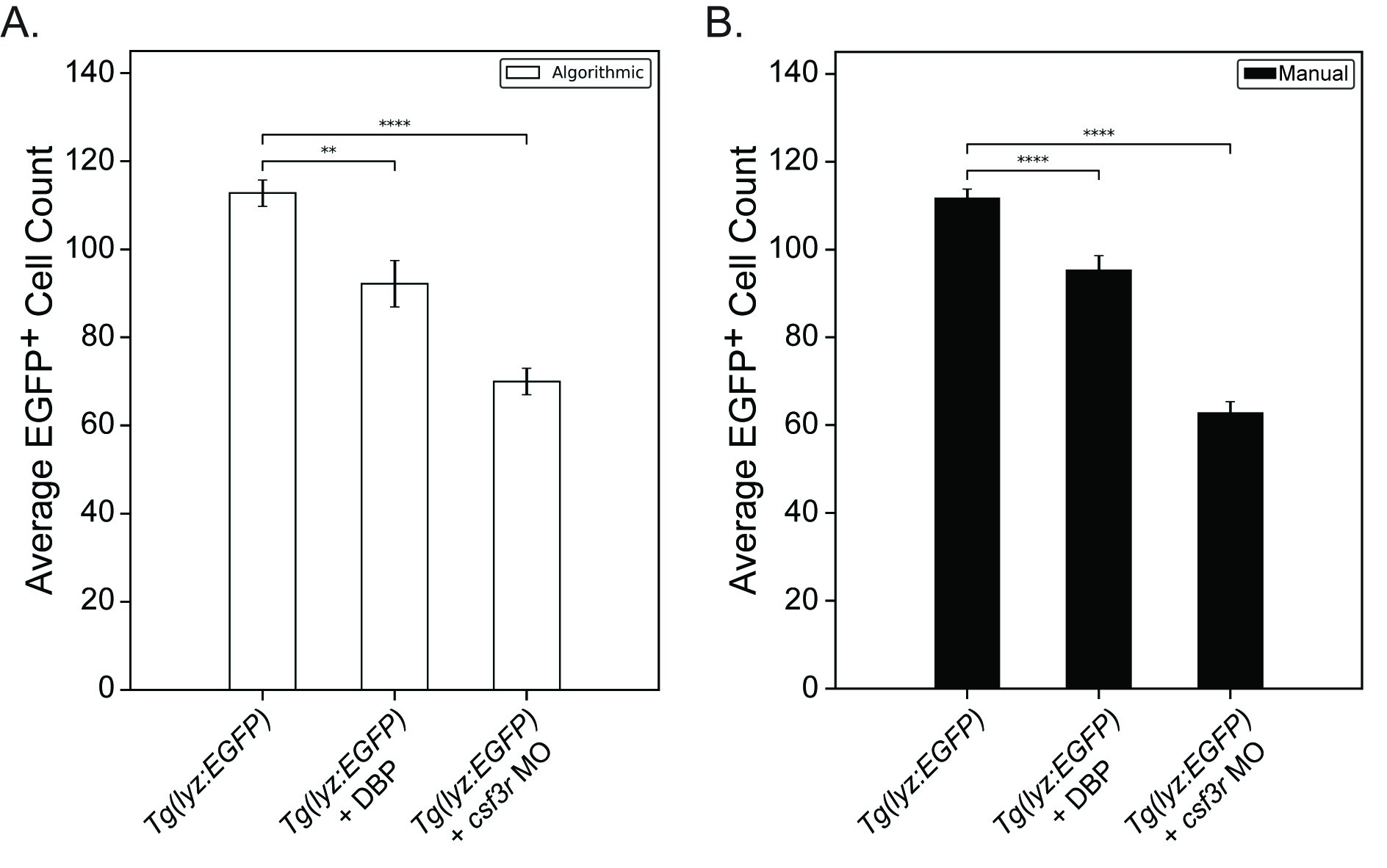

Supplement: S2 Fig — Knockdown and chemical modulation of zebrafish neutrophil counts. A csf3r antisense morpholino (MO) was injected into one-cell stage zebrafish embryos reducing neutrophil counts at 72 hpf (N = 95 larvae). Another subset of zebrafish was treated with 2 μM dibutyl phthalate (DBP), from 6 to 72 hpf, also reducing neutrophil count but by a more subtle degree (N = 23 larvae). Average neutrophil counts were compared to wild-type (WT) fish (N = 96 larvae) and the statistical significance of each method for reducing neutrophil numbers was determined using A) algorithmic counts and B) manual counts. Data points show average neutrophil count and error bars represent the standard error of each experimental group. p-values were computed using a Mann-Whitney U test. ** p < = 0.01; *** p < = 0.001; **** p < = 0.0001. Note: this is the same dataset shown in Fig 4. (TIF) [file pone.0295711.s002.tif]

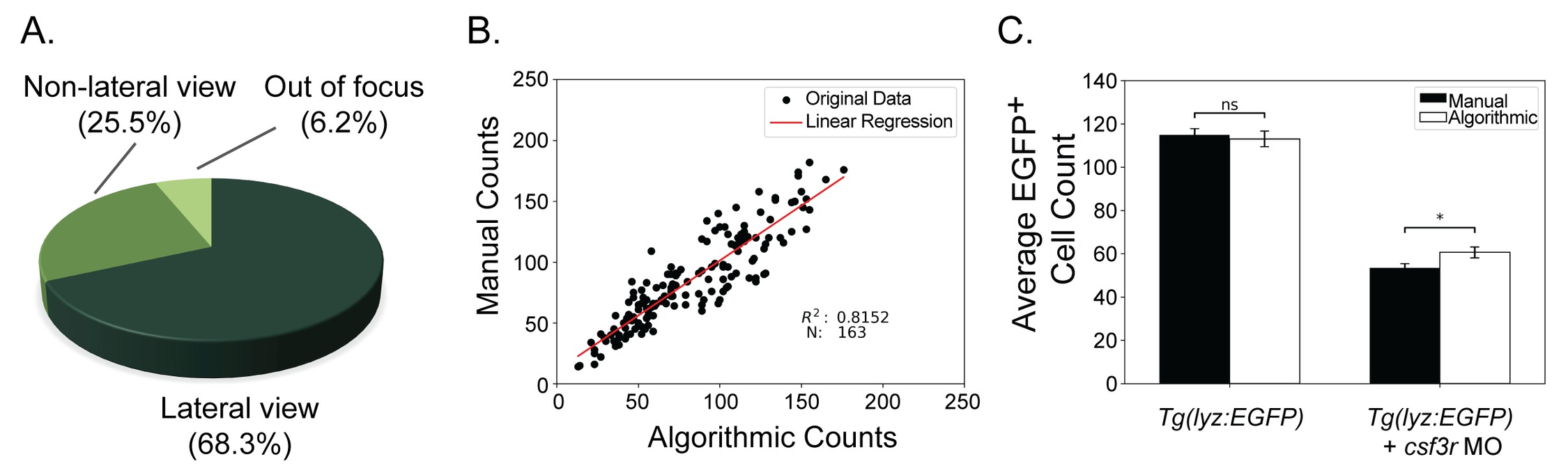

Supplement: S3 Fig — A) Proportion of zebrafish in the lateral or non-lateral orientation in 96-well plates with mesh inserts at 72-hpf (N = 243 larvae). B) Linear regression displaying strong correlation between manual and algorithmic counts for Tg(lyz:EGFP) fish in mesh-well insert well plates (N = 163 larvae). C) Average cell count for untreated Tg(lyz:EGFP) fish in mesh wells (N = 76 larvae) and Tg(lyz:EGFP) fish injected with csf3r morpholino (MO) (N = 87 larvae) as determined by both manual and algorithmic counting. Error bars represent the standard error of each experimental group and p-values were computed using a Mann-Whitney U test. * = p ≤ 0.05, ns = no significance. (TIF) [file pone.0295711.s003.tif]

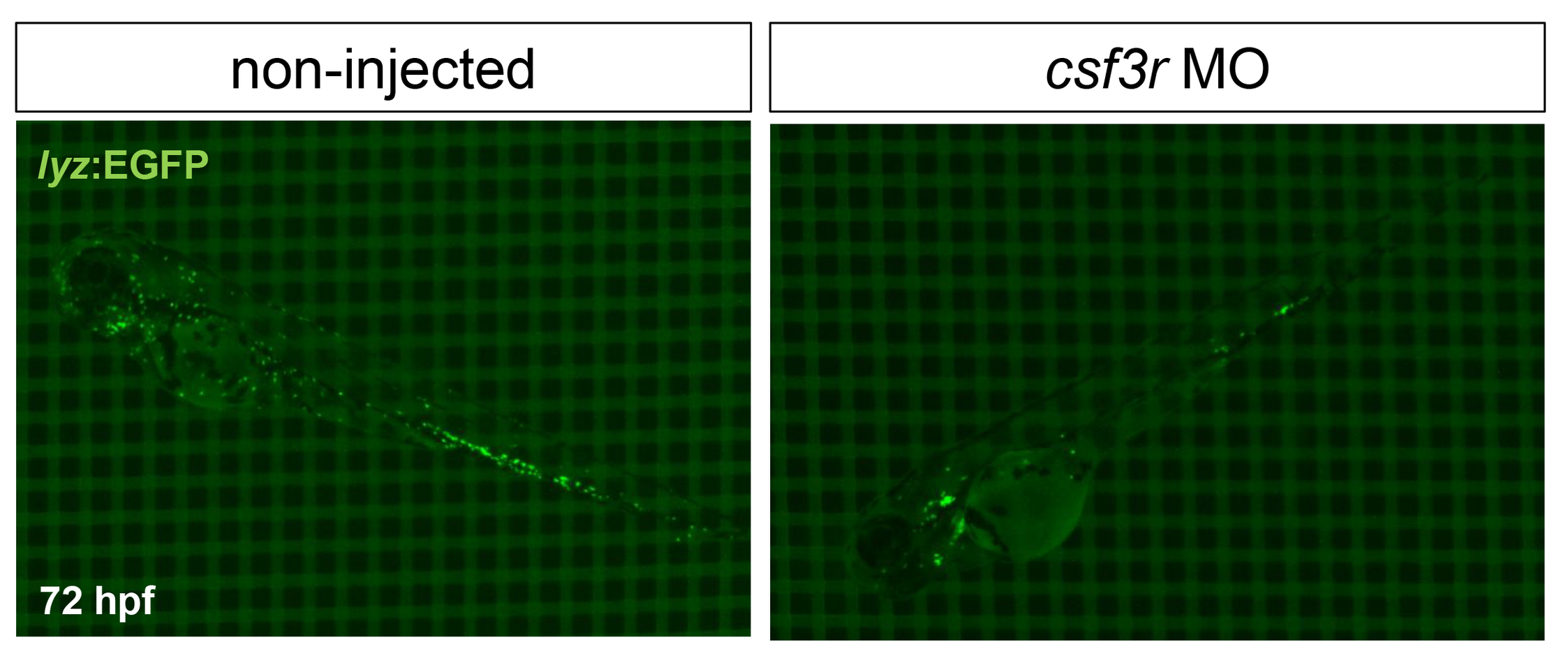

Supplement: S4 Fig — Transgenic Tg(lyz:EGFP) zebrafish larvae (72 hpf) in mesh wells inserts in a 96-well plate. Larve were untreated (left) or injected with a csf3r morpholino (MO) (right). Fish exhibit a lateral orientation defined as having only one eye visible. (TIF) [file pone.0295711.s004.tif]
